# A New Method of Treating Teeth with Diseased Pulps

**Published:** 1888-01

**Authors:** Wilhelm Herbst, L. C. Huyo


ARTICLE II.

### A NEW METHOD OF TREATING TEETH WITH DISEASED PULPS.

BY WILHELM HERBST.

[Read at the Meeting held in Frankfort-on-the-Main, April 30—May 2, 1887.—Translated from the Correspondenz-Blatt fuer Zahnartz, by L. C. Huyo, D. D. S.]

Probably on no one subject connected with dentistry has there been so much said and written as on the treatment of diseased pulps. I take pleasure in presenting a method which I have employed for the past two years with much success. During this time I have saved almost all teeth having aching pulps; and but very seldom has the treatment been attended by untoward sequelæ. I deem it my duty in the interest of our specialty to make known my method, though I know that many a one who swears by root-fillings and antiseptic treatment will incredulously shake his head.

When a patient with an aching tooth comes to me, the pain originating in the pulp, I first syringe the cavity with warm water and carefully remove all extraneous matter and the softened dentine, using cocaine for the palliation of pain. Then, on a pledget of cotton, I take up some cobalt

(*arsenic, metallic, crud.*, moistened with carbolic acid, and lay it on the pulp, covering it carefully with very soft gutta-percha, and taking particular care that there is no undue pressure. I ask the patient to return in two or three days, when I remove the application and enlarge the pulp-chamber with a sharp, rather good sized round bur. After the carious portion of the tooth has been removed, I proceed at once to fill.

The pulp-chamber must be thoroughly syringed with water and dried with punk or bibulous paper. It is then filled with tin, or, beter still, with gold and tin—this to be inserted with considerable pressure by the rotation method * Hereupon the crown cavity proper is filled with gold, gold and tin, cement or amalgam, and, as a rule, the tooth is thoroughly cured.

The cobalt should be kept in a glass phial containing a little carbolic acid. The cotton pledget is dipped into this phial, so that some of the cobalt may be caught up. Before carrying the application to the cavity, the excess of carbolic acid must be soaked up by means of punk or bibulous paper. In drilling out the pulp chamber, one should have a care not to use too small burs, lest the root canals be entered. Under no circumstances must the attempt be made to enter the canals with broaches, excavators, probes, etc., in order to remove the rest of the pulp. For filling the pulp-chamber, I take either tin-foil No. 4 alone, or a sheet of tin-foil No. 4 and one of gold No. 4, so rolled together that the latter may be on the outside. (See *Correspondenz Blatt* for April, 1887, page 120). This combination of foils is placed in the cavity with rather blunt instruments, and then firmly condensed with head-shaped rotation-points, so as to *hermetically seal the chamber*. To this part of the operation I would call especial attention. While enlarging the pulp-chamber, if there should be much sensitiveness, cocaine should be used

---

* Herbst method.—*Translator.*

to secure relief; sometimes it may be necessary even to make another application of cobalt.

The perfect sealing of the chamber can be accomplished by the rotation method only. I regard it as almost impossible with hand pressure or mallet force. For filling the pulp-chamber nothing but tin, or tin and gold, should be used.

It is remarkable that hardly any change of color takes place in teeth treated after the manner described. If it is desired to fill the crown cavity with cement or with gold, the chamber should be filled with tin; but if amalgam is used, gold and tin in combination must be employed, since mercury attacks the tin. Fast-setting amalgams is best here.

Success depends upon hermetically sealing the pulp-chamber.

The question now arises, what becomes of the pulp-stumps? which, as a rule, are alive. According to my opinion, they gradually die, and are disposed of by resorption. I have several times had occasion to examine the root-canals of teeth treated as above. From some cause the amalgam fillings had dropped out; but the seal over the canals had been undisturbed. After applying the rubber dam and drying the teeth, I found that upon removing the tin seal the root canals were *quite empty and odorless.* The pulp-stumps must have died and become resorbed

The operation is most difficult in the distal exposures of molars. There is not much trouble when the grinding surface over the cavities has become partially disintegrated and access can be gained to the pulp chamber by means of chisels or burs; but cavities accessible only by the angle-piece present some difficulty.

Cobalt—the best may be obtained from apothecary Nonne of Bockenheim, near Frankfort-on-the-Main—is a most valuable drug in dentistry and is preferable to the white arsenic. When employing my method, it is important that the treated teeth or their antagonists be somewhat

2

ground off at the occluding surface, and that the filling be not built so high as to receive too much pressure during mastication.

I have waited two years before making public what, in my opinion, is a most important innovation. Many hundreds of teeth have I treated by this method with the greatest of success. I beg that it receive earnest consideration, and that my directions be accurately followed.—*Southern Dental Journal.*

